# Postural Hand Synergies during Environmental Constraint Exploitation

**DOI:** 10.3389/fnbot.2017.00041

**Published:** 2017-08-29

**Authors:** Cosimo Della Santina, Matteo Bianchi, Giuseppe Averta, Simone Ciotti, Visar Arapi, Simone Fani, Edoardo Battaglia, Manuel Giuseppe Catalano, Marco Santello, Antonio Bicchi

**Affiliations:** ^1^Centro E. Piaggio, University of Pisa, Pisa, Italy; ^2^ADVR, Fondazione Istituto Italiano di Tecnologia, Genoa, Italy; ^3^School of Biological and Health Systems Engineering, Ira A. Fulton Schools of Engineering, Arizona State University, Tempe, AZ, United States

**Keywords:** postural synergies, human hand motor control, environment constraint exploitation, tactile perception, grasping

## Abstract

Humans are able to intuitively exploit the shape of an object and environmental constraints to achieve stable grasps and perform dexterous manipulations. In doing that, a vast range of kinematic strategies can be observed. However, in this work we formulate the hypothesis that such ability can be described in terms of a synergistic behavior in the generation of hand postures, i.e., using a reduced set of commonly used kinematic patterns. This is in analogy with previous studies showing the presence of such behavior in different tasks, such as grasping. We investigated this hypothesis in experiments performed by six subjects, who were asked to grasp objects from a flat surface. We quantitatively characterized hand posture behavior from a kinematic perspective, i.e., the hand joint angles, in both pre-shaping and during the interaction with the environment. To determine the role of tactile feedback, we repeated the same experiments but with subjects wearing a rigid shell on the fingertips to reduce cutaneous afferent inputs. Results show the persistence of at least two postural synergies in all the considered experimental conditions and phases. Tactile impairment does not alter significantly the first two synergies, and contact with the environment generates a change only for higher order Principal Components. A good match also arises between the first synergy found in our analysis and the first synergy of grasping as quantified by previous work. The present study is motivated by the interest of learning from the human example, extracting lessons that can be applied in robot design and control. Thus, we conclude with a discussion on implications for robotics of our findings.

## 1 Introduction

The human hand is a remarkably complex system, with many joints, ligaments, muscles, and sensory receptors contributing to its wide dexterity. The control of such an abundance or redundancy is classically referred to as Bernstein’s problem (Bernstein, 1967). Several neuroscientific findings (Mussa-Ivaldi, 1999; Saltiel et al., 2001; Latash, 2008; Overduin et al., 2014; Stratmann et al., 2016) suggest that the human nervous system is able to cope with such complexity leveraging on a control space of reduced dimensionality. Under this regard, synergies were defined as principal patterns of actuation. Through them, the human Central Nervous System, also leveraging upon peripheral constraints, can generate movements by combining pre-organized patterns, i.e., by simultaneously activating different degrees of freedom, instead of acting separately on each joint or muscle. These patterns are implemented at various levels of the motor control architecture, i.e., either they are represented by cortical mechanisms, hard-coded in low-level neural circuits, or generated by the mechanical organization of human musculoskeletal system (Tresch et al., 1999; Santello et al., 2013; Leo et al., 2016). The synergy concept has been investigated in different tasks. Examples regarding hand control are grasping imagined objects (Santello et al., 1998), reach-to-grasp (Mason et al., 2001), and precision grip (Grinyagin et al., 2005).

Another key aspect in the generation of meaningful motor actions was recognized in the interplay between synergistic components and the external environment (Feldman et al., 2015). Indeed, from a sensory point of view, it plays a crucial role to build up our knowledge of the world. Under this regard, a well-known characterization of hand movements during the exploration of the environment is represented by the Exploratory Procedures identified by Lederman and Klatzky (1990). Exploratory Procedures are stereotyped hand motions characteristic of human haptic exploration of the environment. By applying these procedures, subjects try to maximize the amount of haptic information acquired from external objects. Thakur et al. (2008) analyzed Exploratory Procedures through Principal Component Analysis, identifying a reduced set of postural synergies generating the observed behavior.

**Figure 1:**
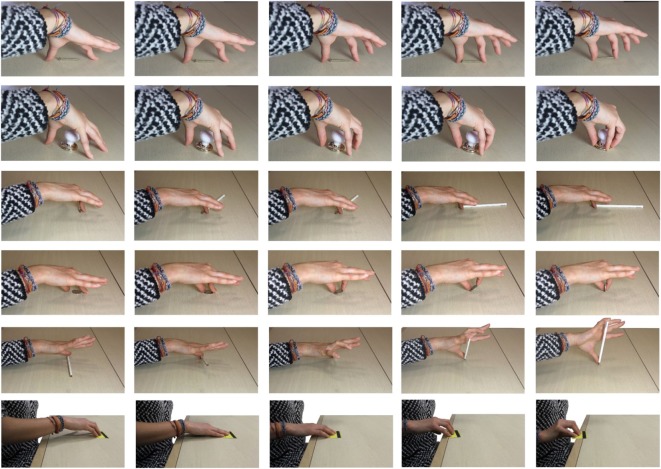
Photo sequences of some examples of experimental constraint exploitation (one for each row). In the first two rows, the subject uses the surface to guide the fingertips position and robustly grasp the object (pinch grasp in the first row, power grasp in the second). In the third row, the subject uses the surface to reorientate the object. In rows four and five, two thin objects are grasped by flipping them using the surface as pivot. In the last row, the subject shifts the object by sliding it through the surface to the edge of the table, where it can be efficiently grasped. From these few examples, we can observe a large variety of strategies and related hand postures.

Focusing instead on the use of constraints for effective motor task accomplishment, Environmental Constraint Exploitation (ECE) primitives are defined in the study of Eppner et al. (2015). We will refer in the following to ECE strategies as actions which aim to manipulate or grasp an object and involve the use of a constraint as central element of the strategy. Figure 1 shows some examples reproducing experimentally observed behaviors, where a subject exploits a flat surface. In Puhlmann et al. (2016), a taxonomy of ECE primitives is proposed. In Eppner et al. (2015), the authors also show that impairing the use of the environment during grasping and manipulation sensibly decreases subjects’ performance, suggesting the central role of these strategies in humans. This way of manipulating and grasping is very different from the classical strategies used in robotics, where the contact with the environment is avoided as much as possible (Bonilla et al., 2014). Thus, it is authors’ opinion that a deeper understanding of ECE can directly affect the performance of robotic manipulators.

In this work, we investigate the presence of a synergistic behavior underlying the generation of hand postures during ECE, execution, and planning. We also investigate the effect that cutaneous impairment might have on such synergies. We were motivated in doing this by the many studies in the literature highlighting the central role of tactile information in grasping (Johansson and Westling, 1984; Westling and Johansson, 1987; Nowak et al., 2004).

We performed experiments with six participants, who were asked to grasp a set of objects from a flat surface. We selected this task since it represents a good trade-off between analytic complexity and richness of kinematic behavior induced by ECE, as accounted, e.g., by Figure 1 and in the study of Eppner et al. (2015). Two experimental conditions were considered: with and without cutaneous impairment. In the first case, participants were requested to wear rigid shells at their fingertips. Results were processed through Principal Component Analysis, as it is common in literature (Santello et al., 2013), in order to check the existence of a set of Principal Components describing the recorded data. We performed the analysis during the actual contact with the environment and on pre-shaping postures, i.e., during the approaching movement that precedes the hand–object–table interaction.

A marked synergistic behavior results in all the considered experiments. Both the interaction with the external environment and the cutaneous impairment appear to modify only the higher order Principal Components. Furthermore, the first synergy exhibits a good resemblance with the one observed in grasping of imagined objects (reported in Santello et al. (1998)).

In recent years, the idea of hand synergies (with special focus on grasping) has been successfully applied in robotics for a simplified yet effective design, sensing, and control of artificial systems. For a detailed review, we refer the interested reader to the studies of Alessandro et al. (2013) and Santello et al. (2016). In accordance with this previous successful experience of mutual inspiration between neuroscience and robotics, we believe that the here proposed results can inform the design and control of artificial hands able to take full advantage of the Exploitation of Environmental Constraints. We discuss these aspects in the final part of the work.

## 2 Materials and Methods

### 2.1 Participants

We tested six able-bodied volunteers (three females, three males; age range: 23–27 years, mean 25.17 years). All subjects were tested on their dominant hand (right hand; self-reported hand dominance). All participants were naive to the experimental purpose of the study and had no history of neuromuscular disorders. Before data collection, subjects signed an informed consent to participate in the experiment. The experimental protocols were approved by the Institutional Review Board of University of Pisa, in accordance to the Declaration of Helsinki.

### 2.2 Apparatus

**Figure 2:**
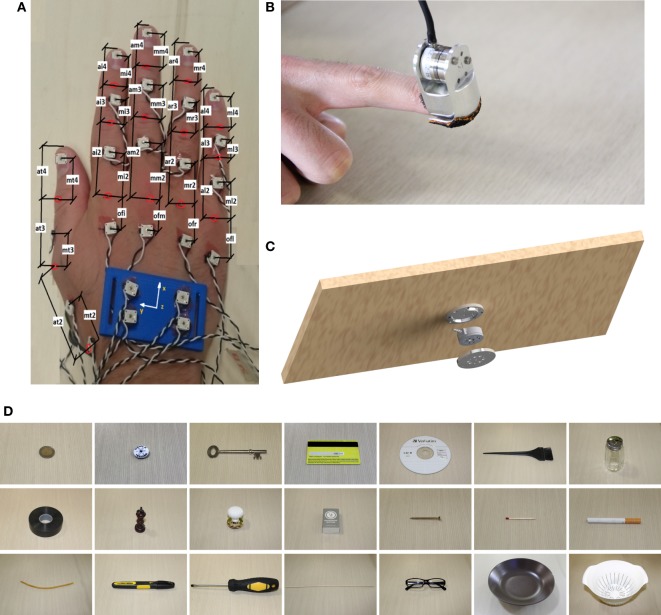
Experimental apparatus. Kinematic measures are kept through an active marker motion capture system. Impaired condition is imposed through ThimbleSenses’ rigid shells. Force and torque data were acquired from the sensorized table. **(A)** LED distribution, **(B)** ThimbleSense, **(C)** sensorized surface, and **(D)** set of objects used in the experiment.

**Figure 3:**
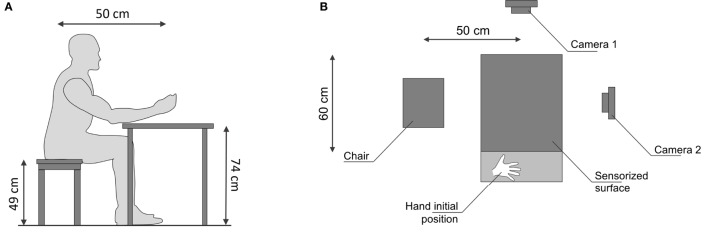
Experimental setup. The subjects sat comfortably in front of the table. In the starting position, subject’s hand was located over the drawing on right side of the sensorized surface. In addition to the ten cameras used for the motion capture, two cameras are included in the setup to record the scene. **(A)** Setup side view. **(B)** Setup from above.

For the present investigation we employed a multimodal acquisition setup (see Figure 2), composed of:

- Phase Space Motion Capture System; we record kinematic data using a commercial system for 3D motion tracking with active LED markers, the Phase Space.^1^ Ten stereo cameras working at 480 Hz tracked 3D positions of 24 markers fastened to hand and phalanges as shown in Figure 2A. The LED frequency is in the visible red.
- A fingertip shell (Figure 2B); we use the shell of ThimbleSense sensor (Battaglia et al., 2016). Subjects wore ThimbleSenses in all fingertips during the tactile impairment experiments, as in Figure 2B. Each shell is connected to the correspondent fingertip as in classical thimbles. Please refer to Battaglia et al. (2016) for a more extensive description of the impairment effects of the shells. Also force information is acquired, to be used in future works. Subjects wore ThimbleSenses on all fingertips, during the tactile impairment experiments.
- A set of 21 objects (Figure 2D); this is composed of 2 euro coin, button badge, key, credit card, CD, comb hair color, salt shaker, tape, chessman (queen), knob, matchbox, screw, match, cigarette, rubber band, marker, screw driver, shashlik, glasses, coffee mug, and plate.
- Sensorized platform (600 mm × 400 mm), which includes the force-torque sensor ATI mini45E mounted as in Figure 2C. It allows the sensing of interaction forces/torques with the table where objects were placed.
- Two cameras (Logitech hd 1080p) to record from two viewpoints the experiments, as in Figure 3B. The recordings were used to visually check the effectiveness of the segmentation. We use blue filters to improve the quality of the recording, which was reduced by the poor light conditions and the red light emittance due to the motion capture system.

In contrast with previous work on grasping real objects (e.g., Mason et al., 2001), we chose objects that are difficult to grasp and therefore necessitate exploitation of the flat surface constraint. A similar set of objects was used in Eppner et al. (2015) in an analogous table-top scenario, demonstrating the effectiveness of eliciting a rich set of ECE strategies.

The acquisition is implemented through a custom application developed in C++ employing:

- Boost libraries (Schäling, 2011) to perform the synchronization between Phase Space data and force/torque sensors.
- Phase Space OWL library to get the optical tracking system data.
- A custom library providing an interface to acquire the force/torque sensor data (Serio et al., 2014).

The acquisition system allows organizing and synchronizing data with respect to an absolute clock with period 0.025 s.

### 2.3 Experiments

The experiments were designed with the aim of identifying kinetostatic primitives used by humans in grasping and manipulation tasks involving ECE. The subject was asked to comfortably seat in front of the table, as in Figure 3A. The subject was instructed to pose her/his hand in the initial hand position location, positioned at the right side of the sensorized surface as in Figure 3B. The distance between the subject’s hand starting position and the site where the object was placed was 60 cm. For each trial, subjects were asked to reach the object posed in the center of the sensorized surface. Once the hand had reached the object, subjects were asked to grasp, lift (~20 cm height), hold (~1 s), put it back on the table, and place the hand back to its starting position. The experimenter gave the starting signal to subjects. In the instructions, the experimenter emphasized that the whole movement should be performed in a natural fashion, i.e., the object should be grasped as if the subject was about to use it, in accordance with Santello et al. (1998). Two trials were performed for any of the 21 objects grasped. The object order was randomized for every subject. The sequence of trials was repeated two times, with and without tactile impairment, for a total of 84 trials per subject. Each subject performed the whole experiment in a single day. The experiments with tactile impairment were performed in the morning, the experiments without tactile impairment in the afternoon, in agreement with previous experiments (Battaglia et al., 2016). Each subject performed the experiment in an independent day w.r.t the other ones.

### 2.4 Data Pre-Processing and Analysis

Data processing is organized in two phases. First, data are pre-processed to reduce the noise and to evaluate joint angles and contact points. Second, we perform statistical analyses to identify the presence of a synergistic behavior in ECE. We perform the analyses in pre-shaping and during the whole contact with the environment. To quantify the role of tactile impairment, we consider separately the impaired and unimpaired cases. The analysis was performed considering the whole group of participants. In Section 3.4, we report the CI for the dot products between the synergies’ vectors.

#### 2.4.1 Data Processing

Raw data collected from the experimental setup were force and torque from the six axes F/T sensor ATImini45 and 24 LED positions from the motion capture system, both with and without tactile impairment.

##### 2.4.1.1 Kinematic Model of the Human Hand

**Figure 4:**
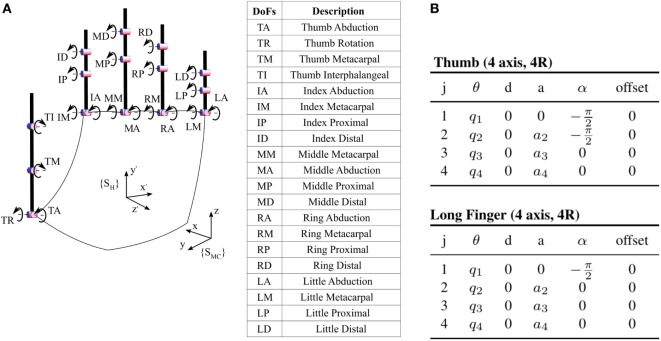
Kinematic model of the human hand considered in this work. The model has 20 DoFs, which include the 15 DoFs of the hand model used in Santello et al. (1998). The left panel graphically describes the kinematics, while the right one specifies the Denavit–Hartenberg parameterization of thumb and the long fingers. We denoted with *j* the joint index, starting from the proximal joint to the distal, while *q*_1_, *q*_2_, *q*_3_, *q*_4_ are the joint angles and *a*_2_, *a*_3_, *a*_4_ are the phalanx lengths. **(A)** Kinematic model. **(B)** DH parameterization.

An accurate description of the human hand is challenging due to the high number of bones and joints composing the human hand. As a trade-off between accuracy and complexity, in this work we considered a 20 DoF kinematic model of the human hand (Figure 4A). Each long finger is described by a set of four angles: two DoFs for flexion–extension and abduction–adduction in metacarpophalangeal joints, one DoF for flexion–extension in proximal and distal intraphalangeal joints. The thumb is described with four angles: two DoFs for the trapeziometacarpal, one DoF for the metacarpophalangeal, and one DoF for the interphalangeal. For the sake of space, we do not report here the mathematical form of the kinematics,^2^ which can be easily derived from the Denavit–Hartenberg parameterization in Figure 4B (see, e.g., Murray et al. (1994)). In the Appendix, we report the direct kinematics derivation for the led positioned on a long finger nail. A key characteristic of the model is that it shares the 15 DoFs of the model used in Santello et al. (1998), allowing an easy comparison with the classical postural synergies of grasp, as done, e.g., in Gabiccini et al. (2013).

##### 2.4.1.2 Model Calibration

To reconstruct realistic values of the joint angles from marker data, the kinematic model employed should reproduce as closely as possible the actual kinematics of the subject being recorded. To achieve this goal, we implement an identification procedure that follows what was done in Gabiccini et al. (2013). The data set is acquired by asking the subject to perform the Kapandji test (Kapandji, 1985), i.e., touching the four long fingers with the tip of the thumb. The test is repeated two times.

The parameters to be identified are: $a_{\text{B}}\in R^{30}$ collecting the length of each phalanx and the three space positions of the abduction joints (TR/TA, IM/IA, MM/MA, RM/RA, and LM/LA in Figure 4A) w.r.t. the local frame, and $a_{\text{G}}\in R^{45}$ collecting the location of each marker placed on the phalanxes w.r.t. the joint to which the LED is connected.

Their values are evaluated as ones minimizing the Root Mean Square Error (RMSE) between measured marked positions *y_k_* and the estimated ones from the hand kinematics *f* (*x_k_*; *a_G_*, *a_B_*), in a set of reference time stamps $k_{\text{id}}$

(1) $$\begin{array}{l} \text{arg}\;\underset{x,a_{\text{G}},a_{\text{B}}}{\text{min}}\sqrt{\sum_{k\in K_{\text{id}}}\;{\left(y_{k}-f\left(x_{k};a_{\text{G}},a_{\text{B}}\right)\right)}^{T}\left(y_{k}-f\left(x_{k};a_{\text{G}},a_{\text{B}}\right)\right)}\\ \text{s}.\text{t}.\;x_{k}>0\;\forall k, \end{array}$$

where $x_{k}\in R^{20}$ is the vector collecting the estimation of joint angles at the *k*th time step, *x_k_* > 0 is to be considered element-wise, *x* is the vector collecting all the joint estimations in the considered time steps. We selected $k_{\text{id}}$ as 60 equidistant frames. The constraint *x_k_* > 0 accounts for biomechanical joint limits, in order to achieve a more robust estimation.

The optimization problem is solved through the MatLab function *fmincon*. The initial guess for the parameters *a_G_* and *a_B_* are evaluated through direct measurements, done with a caliper on each subject’s hand before the experiment, while the initial value of *x* is the null vector. Since we do not have direct access to the real joint angles, the quality of the calibration is evaluated as the RMSE between the measured led positions, and the ones resulting from the identification, as, e.g., in Gabiccini et al. (2013). The average error across trials and subjects is 2.2 mm for the unimpaired case and 3.1 mm for the impaired case.

##### 2.4.1.3 Joint Angles Estimation

Articulated hand postures typically produce marker occlusion to cameras. We use here Piecewise Cubic Hermite Interpolating Polynomials (De Boor et al., 1978) to interpolate missing values. To estimate the hand postures from marker position we propose a Constrained Extended Kalman Filter (Sircoulomb et al., 2008). Kalman filtering was already employed for a similar scope in Fu and Santello (2010). We rely on the identified hand model, representing joint evolution as a random walk. In each time step, the filter estimates the hand posture from the measure of the marker position in space and previous state estimation. Joint limits are considered as constraints. To increase the robustness against marker losses (we had an average of 2.47 marker loss per frame, and a maximum number of consecutive marker loss of ~15), we multiply at each step the Kalman Filter observation noise covariance matrix by the number of consecutive missing measures. The filter is initialized with the open hand posture and a null state covariance matrix. The observation noise covariance matrix is 0.001 *I*_20×20_, and the state noise covariance matrix is 0.0005 *I*_24×24_. Both the matrices are heuristically tuned. The quality of the reconstruction is evaluated as the RMSE between measured and estimated LED positions. The mean values are 2.9 mm for the unimpaired case and 3.2 mm for the impaired case.

##### 2.4.1.4 Force Data

The force/torque data from ATI Mini45 (i.e., sensorized surface) are filtered through a moving average filter based on the Savitzky–Golay method (Schafer, 2011). The window width is heuristically tuned as the 1.5% of total data length. We then use the knowledge of surface form, to evaluate the centroid of contact of Force/Torque data.^3^

#### 2.4.2 Analysis

**Figure 5:**
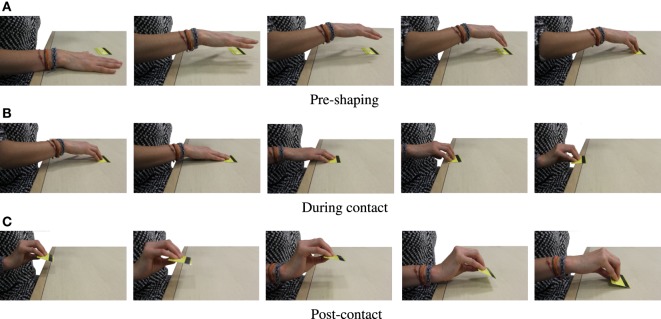
Photosequences of experimental constraint exploitation. According to our contact-based classification, **(A)** presents the *pre-shaping* phase, **(B)** shows the *during contact* phase, and **(C)** the *post-contact* phase.

We segment pre-processed data into three main phases, also described in Figure 5: (i) pre-shaping, where the object is reached and the hand posture is shaped in order to purposefully interact with the environment, (ii) contact, in which the constraint is exploited in order to manipulate and grasp the object, and (iii) post-contact, when the object is grasped and lifted from the table.

By considering non-adhesive interactions with the environment, we can assume any change occurring to the force orthogonal to the surface as due to an interaction. We thus segmented the actions searching for a change in the corresponding force measured by the sensorized surface (see Section 2.2). The cutoff from the first and the second phase is identified by the first contact with the table, when the force starts to increase. To accurately detect this point we consider both to the signal and to its derivative. The cutoff identifying the end of the contact phase is taken as the first time in which the contact force returns to zero.

The aim of the analysis is to identify a subspace of reduced dimensionality embedding the hand postures, to test the hypothesis of presence of a synergistic behavior in Environmental Constraint Exploitation. Principal Component Analysis (PCA) is a valuable tool to achieve this goal (Santello et al., 1998, 2013; Mason et al., 2001; Thakur et al., 2008). Given a set of data, described by a correlation matrix *C* and a mean *m*, PCA derives an orthonormal base of the data space, whose first element *S*_1_ indicates the direction where data present the greatest variability. In turn, each successive component *S_i_* has the highest variability under orthogonality constraint. The *i*th element of the base is referred to as Principal Component of the data set. The normalized percentage of the data variability projected on each Principal Component is called explained variance of the component.

We evaluate PCA as the singular value decomposition of the data correlation matrix *C*, i.e., by finding an orthonormal matrix Σ, which brings *C* in Jordan form through the similitude transformation Σ*^T^C* Σ. In that case, the Principal Components are the column of the matrix Σ = [*S*_1_, …, *S_n_*], and the explained variances are the corresponding eigenvalues. For further details on PCA we refer the interested reader to the study of Jolliffe (2002).

If PCA is used to analyze hand postures in joint space, explained variances can be used to understand if the hand moves on a reduced set of the configurations by looking if there are few principal directions that explain the major part of the data. If this is the case we refer to such principal directions as synergies.

It is worth noticing that the use of the same calibrated kinematic model for every subject enables a coherent description of the hand configuration space as $R^{20}$ for all the experimental conditions. We consider cosine of the angle between synergy’s directions as the metric to compare results of the different analyses. We evaluate it as the absolute value of the normalized dot product between the synergy vectors.

We also compare synergies resulting from our analysis with those employed for the execution of grasping, as shown in Santello et al. (1998). The kinematic model in Santello et al. (1998) takes into account a subset of the joints considered in this work (see Section 2.4.1.1). Thus, the configuration space of grasping synergies is a subset of dimension 15 of the hand configuration space considered in this work. To compare vectors, we projected full hand configurations in the corresponding subspace. This is equivalent to simply neglect the values corresponding to joints ID, MD, RD, and LD in Figure 4A.

Since our main focus is to extract lessons that can inform the robotic design (as discussed in Section 4.2), a complete study of inference is beyond the scope of the present work. However, we considered Student’s *t* test to infer the general validity of some of our results. Please refer to the study of Hogg and Craig (1995) for an exhaustive introduction.

##### 2.4.2.1 Pre-Shaping Analysis

The pre-shaping analysis is done with the purpose of identifying kinematic regularities in the generation of hand postures for the ECE exploitation. In Santello et al. (1998), PCA is performed for constant postures acquired in grasping. These authors took out effects due to interaction with the objects, by asking subjects to grasp imagined ones. In this analysis, we aim to achieve the same goal by performing PCA on a data set composed of the last poses before the contact with the environment in each trial, i.e., the last pose in pre-shaping phases when a purposeful interaction with the environment is planned. Unimpaired and impaired cases are separately analyzed. Both data sets are composed of 252 poses (six subjects, 21 objects, and two trials).

##### 2.4.2.2 Contact Analysis

In order to evaluate if there are some kinematic regularities in the strategies employed by the subjects during actual exploitation of the surface (see, e.g., Figure 1), we perform PCA on data collected during the contact phase. We perform the analysis separately in the impaired and unimpaired case. To characterize the effect of cutaneous impairment we also evaluated the mean amount of time in which subjects stay in contact with the table, mean time for task accomplishment, and the mean norm of interaction forces, by averaging the correspondent values for every subject and every object.

The data sets for both impaired and unimpaired conditions are composed of a variable number of poses depending on the strategy execution time. Each ECE generates an amount of postures equal to 40 times the execution time (see acquisition rate in Section 2.2). All 21 objects, two trials, and six subjects were considered.

##### 2.4.2.3 Differences between Pre and during Contact

We investigate the persistence of the same basic ingredients of hand posture before and during the contact with the environment for both bare fingers and cutaneous impairment conditions. The dot product of synergies evaluated in previous sections was computed in order to quantify their similarities.

## 3 Results

### 3.1 Pre-Shaping Analysis

**Figure 6:**
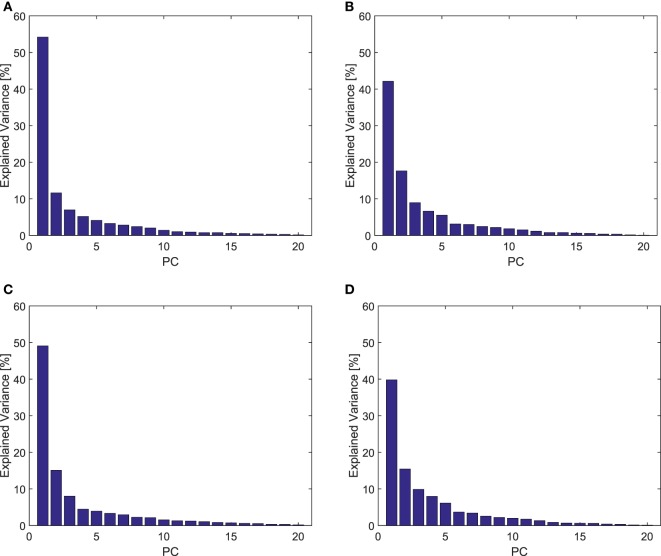
Explained variance resulting from PCA analysis of postures in pre-shaping and during contact, in tactile impaired and unimpaired case. A marked predominance of the first Principal Component is present in all the cases, showing a synergistic behavior. **(A)** Unimpaired, pre-shape; **(B)** impaired, pre-shape; **(C)** unimpaired, contact; and **(D)** impaired, contact.

**Figure 7:**
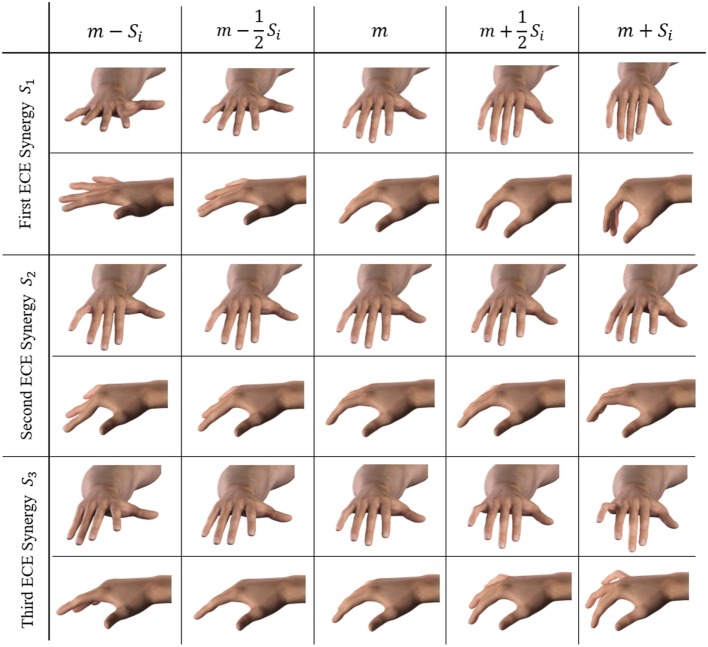
Graphical representation of the movements w.r.t. the mean hand posture, associated with the first three synergies during pre-shaping in unimpaired condition. Each column presents a different stage of the synergistic movement, obtained by summing the hand mean configuration *m*, to the synergy vector *S_i_* of the *i*th synergy.

**Figure 8:**
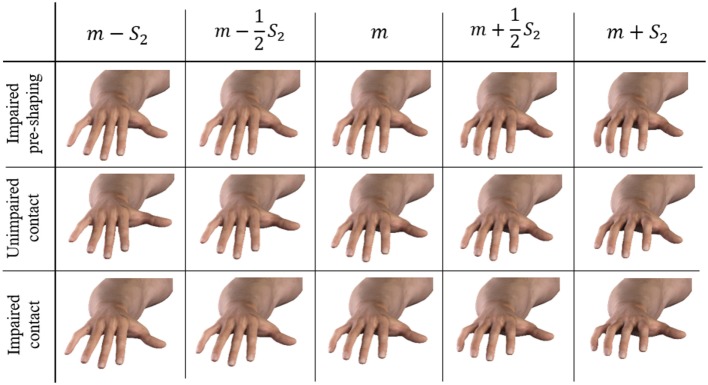
Graphical representation of the movements associated with the second ECE synergy. Pre-shaping impaired, contact unimpaired, and contact impaired conditions are considered. The mean posture is referred to as *m*, the second synergy as *S*_2_. We do not report here the first synergies, since they do not present visible discrepancies from each other. The present figure shows also a good coherence in the behavior described by the second synergy, among the considered conditions.

Figure 6 shows the explained variance associated with the PCs on the poses during the pre-shaping phase. For the unimpaired case, the first Principal Component explains about 54% of the variance, while the first three pre-shaping synergies explain more than 72%. Figure 7 shows the graphical representation of the first three resulting postural synergies. The same analysis in the impaired case shows that the first synergy explains about 42% of the variance, while three Principal Components explain more than 68% of the variance. Thus, for our data set, the variance explained by the first main synergies is lower in the impaired condition. In Figure 7, we present the graphical representations of the movements corresponding to the first three synergies of pre-shaping without tactile impairment. Table 1 presents the numerical values of the first ECE synergy of pre-shaping with and without impairment, in comparison with the first synergy of grasp (Santello et al., 1998). Figure 8 presents the movement corresponding to the second synergy of pre-shaping with tactile impairment.

**Table 1:** Numerical values of the first synergy of Grasp (Santello et al., 1998) and of Environmental Constraint Exploitation, with and without impairment, before and after contact.

DoFs	Grasp	Unimpaired pre-shape	Impaired pre-shape	Unimpaired contact	Impaired contact
TA	−0.43	−0.14	−0.15	−0.12	−0.15
TR	0.29	0.31	0.35	0.30	0.34
TM	0.14	0.14	0.17	0.17	0.16
TI	0.03	0.04	0.05	0.05	0.09
IA	−0.13	−0.08	−0.12	−0.08	−0.11
IM	0.33	0.39	0.35	0.40	0.34
IP	0.15	0.16	0.16	0.17	0.20
ID	x	0.01	0.03	0.03	0.05
MA	x	−0.03	−0.08	−0.02	−0.06
MM	0.33	0.38	0.33	0.38	0.32
MP	0.16	0.27	0.27	0.27	0.30
MD	x	0.04	0.06	0.05	0.08
RA	0.06	0.00	−0.02	0.02	−0.02
RM	0.40	0.44	0.37	0.43	0.32
RP	0.20	0.22	0.27	0.22	0.35
RD	x	0.04	0.06	0.05	0.11
LA	0.14	0.05	0.1	0.08	0.09
LM	0.37	0.43	0.42	0.41	0.37
LP	0.27	0.12	0.21	0.17	0.24
LD	x	0.02	0.04	0.02	0.08

*We indicate with “x” the DoFs that were not considered in Santello et al. (1998)*.

In all the considered cases, the first synergy describes an opening–closing behavior of the whole hand, while the second synergy corresponds to a closure of the distal joints mostly of index and medial fingers and a closing of the thumb. The third synergy is similar to the second, but concerning the little and ring fingers.

**Figure 9:**
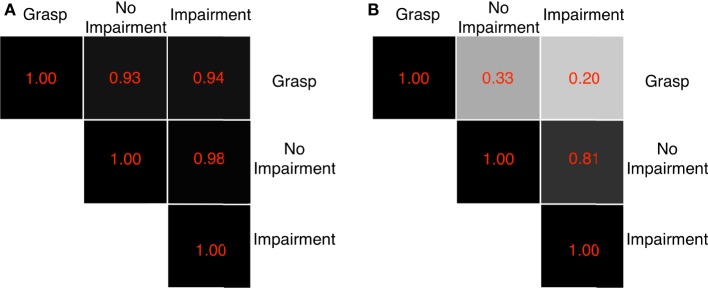
Dot products between first and second grasp synergies and first and second ECE synergies evaluated during pre-shaping, with and without tactile impairment. The gray scale graphically codes the product value: black is 1, i.e., very similar, white is 0, i.e., very different. A high correlation between the first grasping and ECE synergies is evidenced. The ECE synergies with and without impairment present high similarity, which, however, drops for higher order synergies. **(A)** First synergies and **(B)** second synergies.

Figure 9A shows the scalar product between the first grasp synergy in Santello et al. (1998) and the ones found in this work for both sensory conditions. What is noticeable is that there is a high level of consistency between the main synergy of grasping and pre-shaping of human hand in impaired and unimpaired conditions (≥0.9). The similarity is reduced for the second synergy, as shown in Figure 9B, and so on for the other orders. Figure 9 reports also a high correlation between pre-shaping synergies with and without tactile impairment. The presence of impairment does not alter the first two synergies during the pre-shaping phase. However, the similarity strongly drops when the synergy order increases further, reaching 0.36 for the third and 0.003 for the forth.

### 3.2 Contact Analysis

In the unimpaired condition, the first Principal Component explains about the 49% of the variance, and the first three synergies more than 73%. The same analysis in the impaired case returns a first synergy explaining about the 39% of the variance, and the three Principal Components explaining more than 65% of the variance. Also in this case, for our data set the percentage of variance explained in the impaired condition is lower. Table 1 presents the numerical values of first ECE synergy during the contact with the environment, with and without impairment, in comparison with the first synergy of grasp (Santello et al., 1998). Figure 8 presents the movement corresponding to the second synergy with and without tactile impairment.

The analysis also demonstrates that subjects with tactile impairment are in contact with the table for an average time of 4.2 ± 3.1 s, while for the unimpaired case the average time is 2.4 ± 2.4 s. The complete task is performed in 13.9 ± 2.7 s for the tactile impairment case, while it is performed in 11.5 ± 2.0 s in the other case. Finally, the contact force is different for the experiments considered. Mean value of norm of contact forces for the tactile impairment case is 23.2 ± 8.6 N, while mean value is 12.3 ± 5.7 N in the other case.

### 3.3 Differences between Pre and during Contact

**Figure 10:**
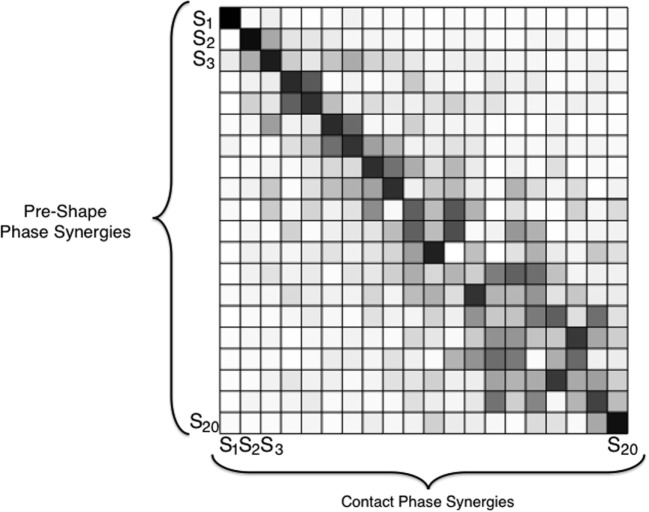
Dot products between the ECE synergies in pre-shaping and in contact with the environment, in the bare finger case. The gray scale graphically codes the product value: black is 1, i.e., very similar, white is 0, i.e., very different. A tendency to maintain the first main components before and after the contact results clearly from this analysis. The results for the impaired case are analogous.

The result shows that the first Principal Components in unimpaired and impaired conditions are very similar w.r.t. the corresponding ones of the pre-shaping analysis. The similarity tends to decrease with the increase in synergy order (correlation ≥ 0.75 till 9th synergy). Dot products are graphically reported in Figure 10 for the unimpaired case. Thus, ECs induce only changes for the high-order synergies, leaving unaltered the main ones, regardless of availability of tactile input. Note that the first synergy is still equivalent to the one in Santello et al. (1998).

### 3.4 Inference and Statistical Relevance

In the previous sections, we presented results from six subjects. Despite such a moderate number of participants, findings and analyses are in line with the existing literature in the field, see e.g., Santello et al. (1998); Mason et al. (2001); and Naceri et al. (2016). To generalize, we here report additional statistical analyses that provide Student’s *t* test based confidence intervals (CI) with 95% probability. CI refers to dot products performed on the PCs extracted for the different conditions and the ones obtained in grasping.

CI for the dot product between the first synergy for impaired and unimpaired conditions is [0.81, 0.96]; CI for the dot product between the first grasping synergy and the unimpaired first ECE synergy is [0.88, 0.94]; CI for the dot product between the first grasping synergy and the impaired first ECE synergy is [0.79, 0.9]. Regarding the contact analysis of Section 3.3, the dot product between the first Principal Component before and during contact results in a CI of [0.97, 0.99] (unimpaired case).

## 4 Discussion and Conclusion

### 4.1 Implications for Motor Control

The explained variance reported in Figure 6 suggests the presence of an underlying synergistic behavior in the purposeful exploitation of environmental constraints. Indeed, the dimensionality of the space required to approximate hand posture is considerably smaller than the number of degrees of freedom. In particular, the first Principal Component shows a marked predominance, with a maximum total variance explained of 54% in the unimpaired pre-shaping case. The three first synergies explain more than 65% of the total variance in all the considered conditions. In the considered experiments, during contact with the environment, the amount of variability accounted by higher order synergies increases. This could be explained by observing that the interaction can shape the subject hand beyond its nominal kinematics. This behavior is also in agreement with the findings in Santello et al. (2002), where the synergistic analysis of Santello et al. (1998) was performed on grasped real objects instead of imagined ones.

Despite our inference analysis (Section 3.4) is limited to the first synergy, a series of characteristics of our data set can be pointed out, which are in accordance with existing neuroscientific findings. Future works will focus on different experimental procedures and tasks to further investigations. In our data set, the first two synergies in the impaired and unimpaired conditions are very similar, as shown in Figures 8 and 9. This suggests that the presence of tactile impairment, while modifying the strategies themselves, does not substantially modify the most basic kinematic ingredients commonly used to generate hand postures. Subjects are aware of the presence of the tactile impairment, so it is reasonable to expect that they might have changed their planning in accordance to that. Indeed, the drop of such similarity after the third synergy suggests that cutaneous impairment affects posture refinement, which can be likely ascribed to higher order synergies (as described in Santello et al. (1998)). However, the amount of data collected does not allow a sound statistical characterization of this behavior. To assess whether higher order synergies are primarily noise or they actually contribute to hand postures, we will resort to the usage of discriminant analysis and information theory, as in Santello et al. (1998), as future works.

Analogously, Figure 10 shows a high similarity in the first Principal Components during contact and pre-shaping, while differences can be observed for higher order synergies. Uncontrolled Manifold theory (Scholz and Schöner, 1999; Latash et al., 2007) suggests that the central nervous system selects in the space of joint angles a subset of variables of interest, which are regulated, purposefully leaving free the remaining variables. The persistence of main postural synergies of pre-shaping during the contact with the environment can be interpreted in the light of this theory by considering the first set of ECE synergies as the variables of interest for the considered task, which remain constant when an external disturbance occurs. The subspace individuated by the higher order synergies is instead left free to adapt to the external environment. Moreover, such behavior could also be due to peripheral constraints embedded in the musculoskeletal system, as discussed, e.g., in Santello et al. (2013).

Data shown in Figure 9 and Table 1 demonstrate that a strong resemblance exists between the first synergy, resulting from the analysis of ECE strategies, and the first synergy of grasp as found in Santello et al. (1998).

This could suggest the presence of underlying synergies, which are integrated with task specific ones. This was proposed, e.g., in Gorniak et al. (2007), and it is in agreement with experimental results presented in Thakur et al. (2008), where task independent synergies are estimated by a set of unconstrained tasks (see CI estimates in Section 3.4).

### 4.2 Implications for Robotics

As it can be widely observed in the literature, neuroscientific results of synergistic behavior of human hands have been successfully translated and applied to robotics to inform the design, control, and sensing of artificial systems, with special focus on grasping, see Bicchi et al. (2011) and Bianchi and Moscatelli (2016). One of the first notable applications of synergies to robotics was in Brown and Asada (2007), where authors propose to use grasp synergies to derive actuation patterns for an underactuated robotic hand. In Gabiccini et al. (2011), the use of hand synergies for the choice of grasping forces is discussed. In Ciocarlie et al. (2007) and later in Amor et al. (2012); Malhotra et al. (2012); and Villani et al. (2012), a synergy based low-dimensional synergistic space is considered to obtain effective pre-grasp shapes for fully actuated robotic hands.

Recently, synergy-inspired actuation has been combined with the introduction of compliance in the structure (Catalano et al., 2014; Xu et al., 2014; Della Santina et al., 2015; Chen and Xiong, 2016) (according to the soft synergy framework (Bicchi et al., 2011)). The availability of robotic hands embedding elasticity in their mechanics has also led to a shift in their control philosophy, accounted, e.g., in Bonilla et al. (2014) and Eppner and Brock (2015). In the classical planning, suitable points are selected on the object to be grasped or manipulated, generating a nominal grasp of good quality. Trajectories are then executed, to correctly position the fingertips while avoiding contacts with the environment. On the contrary, soft manipulation has changed this scheme. The hand-environment contacts are no more avoided but exploited to successfully shape the hand around the object. Under this regard, the study of environmental exploitation in humans could inform the design, planning, and control of robotic hands to take full advantage from the external environment.

The most direct implication of the presented results could leverage upon the observation that the first synergy of grasping is very similar to the first synergy for Environmental Constraint Exploitation (ECE). Thus, the implementation of the first synergy of grasp as degree of actuation can target the twofold goal of realizing underactuated robotic hands that can effectively grasp objects and, at the same time, are able to exploit Environmental Constraints. To increase hand functionalities beyond the first degree of actuation, we could implement additional ECE synergies, possibly in combination with the grasp synergies. For some examples of the implementation of synergies for the design of underactuated robotic hands, we refer the interested reader to Grioli et al. (2012); Della Santina et al. (2015); and Piazza et al. (2016).

Looking at the differences between the impaired and unimpaired conditions, the key kinematic ingredients seem to remain unaltered at least for the gross movements. However, the time for task accomplishment and the force exchanged with the environment is higher for the impaired case. This result could indicate possible sensing strategies for soft robotic hands, i.e., to detect contact with the environment, e.g., through IMU sensors, which can lead to the development of planning and control laws aiming at minimizing force execution on external objects.

## Ethics Statement

Before data collection, subjects signed an informed consent to participate in the experiment. The experimental protocols were approved by the Institutional Review Board of University of Pisa.

## Author Contributions

CS, MB, MS, and AB designed the study. CS, MB, GA, SC, VA, SF, and EB performed the experiments and data analysis. CS, MB, SC, SF, EB, and MC designed and developed the experimental setup. All the authors contributed to writing the manuscript.

## Conflict of Interest Statement

The authors declare that the research was conducted in the absence of any commercial or financial relationships that could be construed as a potential conflict of interest.

## Footnotes

^1^http://www.phasespace.com/, website visited on April 2017.

^2^Note that here we use the term kinematics as it is commonly done in robotics and biomechanics, to refer to the mapping of Lagrangian variables to a different point of the structure. In this context, the evolution in time is referred to as differential kinematics.

^3^The contact points are computed through tactile toolbox, as in Serio et al. (2014). A portable version of this tool as well as raw data of our experiments will be released through handcorpus.

## Abbreviations

DOF, degree of freedom; EC, Environmental Constraint; ECE, Environmental Constraint Exploitation; PC, Principal Component; RMSE, Root Mean Square Error; CI, confidence interval.

## A Appendix

### A.1 Finger Kinematics

The forward kinematics of a serial chain with *n* degrees of freedom is described by post-multiplicating different rotation-translation matrices with the following form

$$T_{i-1,i}=\left(\begin{array}{cc} R_{i-1,i} & h_{i-1,i}\\ 0_{1\times 3} & 1 \end{array}\right),$$

where *R_i_*_−1,_*_i_* is the rotation matrix between frame *i* − 1 and frame *i* and *h_i_*_−1,_*_i_* is the translation vector between frame *i* − 1 and frame *i*. Then the whole forward kinematics is calculated as

$$T_{0,n}=T_{0,1}T_{1,2}\ldots T_{n-1,n}.$$

For the long finger (its kinematics is introduced in section 2.4.1.1 and parametrized with Denavit-Hartenberg in Figure 4B) we have

$$T_{01}=\left(\begin{array}{cccc} C_{1} & 0 & -S_{1} & 0\\ S_{1} & 0 & C_{1} & 0\\ 0 & -1 & 0 & 0\\ 0 & 0 & 0 & 1 \end{array}\right),T_{12}=\left(\begin{array}{cccc} C_{2} & -S_{2} & 0 & a_{2}C_{2}\\ S_{2} & C_{2} & 0 & a_{2}S_{2}\\ 0 & 0 & 1 & 0\\ 0 & 0 & 0 & 1 \end{array}\right),$$

$$T_{23}=\left(\begin{array}{cccc} C_{3} & -S_{3} & 0 & a_{3}C_{3}\\ S_{3} & C_{3} & 0 & a_{3}S_{3}\\ 0 & 0 & 1 & 0\\ 0 & 0 & 0 & 1 \end{array}\right),T_{34}=\left(\begin{array}{cccc} C_{4} & -S_{4} & 0 & a_{4}C_{4}\\ S_{4} & C_{4} & 0 & a_{4}S_{4}\\ 0 & 0 & 1 & 0\\ 0 & 0 & 0 & 1 \end{array}\right).$$

where *C_i_* stands for cos(*q_i_*), *S_i_* stands for sin(*q_i_*), *a_i_* is the length of the phalanx *i* − 1 and *q_i_* is the *i*th joint rotation. Then, the forward kinematics is obtained as

$$T_{04}=T_{01}T_{12}T_{23}T_{34}=\left(\begin{array}{cc} R_{0,4} & h_{0,4}\\ 0_{1\times 3} & 1 \end{array}\right).$$

The extended expression of *T*_04_ is neglected for simplicity and can be easily calculated by the reader. The final position ${\left(\begin{array}{ccc} x_{\mathrm{LF}} & y_{\mathrm{LF}} & z_{\mathrm{LF}} \end{array}\right)}^{T}$ of the external phalanges can be extracted from *T*_04_ by selecting *h*_0,4_:

$$\left(\begin{array}{c} x_{\mathrm{LF}}\\ y_{\mathrm{LF}}\\ z_{\mathrm{LF}} \end{array}\right)=\left(\begin{array}{c} C_{1}\left(a_{2}C_{2}+a_{3}C_{23}+a_{4}C_{234}\right)\\ S_{1}\left(a_{2}C_{2}+a_{3}C_{23}+a_{4}C_{234}\right)\\ -a_{2}S_{2}-a_{3}S_{23}-a_{4}S_{234} \end{array}\right),$$

where *C*_23_ stands for cos(*q*_2_ + *q*_3_), *C*_234_ stands for cos(*q*_2_ + *q*_3_ + *q*_4_), *S*_23_ stands for sin(*q*_2_ + *q*_3_), *S*_234_ stands for sin(*q*_2_ + *q*_3_ + *q*_4_). The same procedure can lead to the description of the thumb forward kinematics. We have

$$T_{01}=\left(\begin{array}{cccc} C_{1} & 0 & -S_{1} & 0\\ S_{1} & 0 & C_{1} & 0\\ 0 & -1 & 0 & 0\\ 0 & 0 & 0 & 1 \end{array}\right),T_{12}=\left(\begin{array}{cccc} C_{2} & 0 & -S_{2} & a_{2}C_{2}\\ S_{2} & 0 & C_{2} & a_{2}S_{2}\\ 0 & -1 & 0 & 0\\ 0 & 0 & 0 & 1 \end{array}\right),$$

$$T_{23}=\left(\begin{array}{cccc} C_{3} & -S_{3} & 0 & a_{3}C_{3}\\ S_{3} & C_{3} & 0 & a_{3}S_{3}\\ 0 & 0 & 1 & 0\\ 0 & 0 & 0 & 1 \end{array}\right),T_{34}=\left(\begin{array}{cccc} C_{4} & -S_{4} & 0 & a_{4}C_{4}\\ S_{4} & C_{4} & 0 & a_{4}S_{4}\\ 0 & 0 & 1 & 0\\ 0 & 0 & 0 & 1 \end{array}\right),$$

and then

(A1) $$\left(\begin{array}{c} x_{T}\\ y_{T}\\ z_{T} \end{array}\right)=\left(\begin{array}{c} a_{4}S_{4}\left(C_{3}S_{1}-C_{1}C_{2}S_{3}\right)+a_{2}C_{1}C_{2}+a_{3}S_{1}S_{3}+a_{4}C_{4}\left(S_{1}S_{3}+C_{1}C_{2}C_{3}\right)+a_{3}C_{1}C_{2}C_{3}\\ a_{2}C_{2}S_{1}-a_{4}S_{4}\left(C_{1}C_{3}+C_{2}S_{1}S_{3}\right)-a_{3}C_{1}S_{3}-a_{4}C_{4}\left(C_{1}S_{3}-C_{2}C_{3}S_{1}\right)+a_{3}C_{2}C_{3}S_{1}\\ a_{4}S_{2}S_{3}S_{4}-a_{3}C_{3}S_{2}-a_{4}C_{3}C_{4}S_{2}-a_{2}S_{2} \end{array}\right).$$

It is worth to be noticed that, while linear w.r.t. the parameters *a*_2_, *a*_3_, *a*_4_, equation (A1) defines a strongly non-linear relationship between joint angles *q*_1_, *q*_2_, *q*_3_, *q*_4_ and LED position.

## References

[B1] Alessandro C. Delis I. Nori F. Panzeri S. Berret B. (2013). Muscle synergies in neuroscience and robotics: from input-space to task-space perspectives. Front. Comput. Neurosci. 7:43. 10.3389/fncom.2013.00043 23626535 PMC3630334

[B2] Amor H. B. Kroemer O. Hillenbrand U. Neumann G. Peters J. (2012). “Generalization of human grasping for multi-fingered robot hands,” in Intelligent Robots and Systems (IROS), 2012 IEEE/RSJ International Conference on (Vilamoura-Algarve: IEEE), 2043–2050.

[B3] Battaglia E. Bianchi M. Altobelli A. Grioli G. Catalano M. G. Serio A. (2016). Thimblesense: a fingertip-wearable tactile sensor for grasp analysis. IEEE Trans. Haptics 9, 121–133. 10.1109/toh.2015.2482478 26462243

[B4] Bernstein N. A. (1967). The Co-Ordination and Regulation of Movements. Oxford, New York: Pergamon Press.

[B5] Bianchi M. Moscatelli A. (2016). Human and Robot Hands: Sensorimotor Synergies to Bridge the Gap Between Neuroscience and Robotics. Springer.

[B6] Bicchi A. Gabiccini M. Santello M. (2011). Modelling natural and artificial hands with synergies. Philos. Trans. R. Soc. Lond. B Biol. Sci. 366, 3153–3161. 10.1098/rstb.2011.0152 21969697 PMC3172595

[B7] Bonilla M. Farnioli E. Piazza C. Catalano M. Grioli G. Garabini M. (2014). “Grasping with soft hands,” in Humanoid Robots (Humanoids), 2014 14th IEEE-RAS International Conference on, Madrid, 581–587.

[B8] Brown C. Y. Asada H. H. (2007). “Inter-finger coordination and postural synergies in robot hands via mechanical implementation of principal components analysis,” in Intelligent Robots and Systems, 2007. IROS 2007. IEEE/RSJ International Conference on (Pittsburgh, PA: IEEE), 2877–2882.

[B9] Catalano M. G. Grioli G. Farnioli E. Serio A. Piazza C. Bicchi A. (2014). Adaptive synergies for the design and control of the pisa/iit softhand. Int. J. Robot. Res. 33, 768–782. 10.1177/0278364913518998

[B10] Chen W. Xiong C. (2016). On adaptive grasp with underactuated anthropomorphic hands. J. Bionic Eng. 13, 59–72. 10.1016/s1672-6529(14)60160-8

[B11] Ciocarlie M. Goldfeder C. Allen P. (2007). “Dexterous grasping via eigengrasps: a low-dimensional approach to a high-complexity problem,” in Robotics: Science and Systems Manipulation Workshop-Sensing and Adapting to the Real World. Available at: https://archive.org/details/sensing_and_adapting_to_the_real_world_2007

[B12] De Boor C. De Boor C. Mathématicien E.-U. De Boor C. De Boor C. (1978). A Practical Guide to Splines, Vol. 27 New York: Springer-Verlag.

[B13] Della Santina C. Grioli G. Catalano M. Brando A. Bicchi A. (2015). “Dexterity augmentation on a synergistic hand: the pisa/iit softhand+,” in Humanoid Robots (Humanoids), 2015 IEEE-RAS 15th International Conference on (Seoul: IEEE), 497–503.

[B14] Eppner C. Brock O. (2015). “Planning grasp strategies that exploit environmental constraints,” in Robotics and Automation (ICRA), 2015 IEEE International Conference on (Seattle, WA: IEEE), 4947–4952.

[B15] Eppner C. Deimel R. Álvarez-Ruiz J. Maertens M. Brock O. (2015). Exploitation of environmental constraints in human and robotic grasping. Int. J. Robot. Res. 34, 1021–1038. 10.1007/978-3-319-28872-7_23

[B16] Feldman A. G. (2015). “Referent control of action and perception,” in Challenging Conventional Theories in Behavioral Neuroscience (New York: Springer). 10.1007/978-1-4939-2736-4

[B17] Fu Q. Santello M. (2010). “Tracking whole hand kinematics using extended kalman filter,” in Engineering in Medicine and Biology Society (EMBC), 2010 Annual International Conference of the IEEE (Buenos Aires: IEEE), 4606–4609. 10.1109/IEMBS.2010.5626513 21096228

[B18] Gabiccini M. Bicchi A. Prattichizzo D. Malvezzi M. (2011). On the role of hand synergies in the optimal choice of grasping forces. Auton. Robots 31, 235 10.1007/s10514-011-9244-1

[B19] Gabiccini M. Stillfried G. Marino H. Bianchi M. (2013). “A data-driven kinematic model of the human hand with soft-tissue artifact compensation mechanism for grasp synergy analysis,” in Intelligent Robots and Systems (IROS), 2013 IEEE/RSJ International Conference on (Tokyo: IEEE), 3738–3745.

[B20] Gorniak S. L. Zatsiorsky V. M. Latash M. L. (2007). Hierarchies of synergies: an example of two-hand, multi-finger tasks. Exp. Brain Res. 179, 167–180. 10.1007/s00221-006-0777-z 17103206 PMC1859846

[B21] Grinyagin I. V. Biryukova E. V. Maier M. A. (2005). Kinematic and dynamic synergies of human precision-grip movements. J. Neurophysiol. 94, 2284–2294. 10.1152/jn.01310.2004 15917316

[B22] Grioli G. Catalano M. Silvestro E. Tono S. Bicchi A. (2012). “Adaptive synergies: an approach to the design of under-actuated robotic hands,” in Intelligent Robots and Systems (IROS), 2012 IEEE/RSJ International Conference on (Vilamoura-Algarve: IEEE), 1251–1256.

[B23] Hogg R. V. Craig A. T. (1995). Introduction to Mathematical Statistics (5th edition). Upper Saddle River, New Jersey: Prentice Hall.

[B24] Johansson R. Westling G. (1984). Roles of glabrous skin receptors and sensorimotor memory in automatic control of precision grip when lifting rougher or more slippery objects. Exp. Brain Res. 56, 550–564. 10.1007/bf00237997 6499981

[B25] Jolliffe I. (2002). Principal Component Analysis. Wiley Online Library.

[B26] Kapandji A. (1985). Clinical test of apposition and counter-apposition of the thumb. Ann. Chir. Main 5, 67–73. 10.1016/S0753-9053(86)80053-9 3963909

[B27] Latash M. L. (2008). Synergy. Oxford University Press

[B28] Latash M. L. Scholz J. P. Schöner G. (2007). Toward a new theory of motor synergies. Motor Control 11, 276–308. 10.1123/mcj.11.3.276 17715460

[B29] Lederman S. J. Klatzky R. L. (1990). Haptic classification of common objects: knowledge-driven exploration. Cogn. Psychol. 22, 421–459. 10.1016/0010-0285(90)90009-s 2253454

[B30] Leo A. Handjaras G. Bianchi M. Marino H. Gabiccini M. Guidi A. (2016). A synergy-based hand control is encoded in human motor cortical areas. Elife 5, e13420. 10.7554/eLife.13420 26880543 PMC4786436

[B31] Malhotra M. Rombokas E. Theodorou E. Todorov E. Matsuoka Y. (2012). “Reduced dimensionality control for the act hand,” in Robotics and Automation (ICRA), 2012 IEEE International Conference on (St. Paul, MN: IEEE), 5117–5122.

[B32] Mason C. R. Gomez J. E. Ebner T. J. (2001). Hand synergies during reach-to-grasp. J. Neurophysiol. 86, 2896–2910. 11731546 10.1152/jn.2001.86.6.2896

[B33] Murray R. M. Li Z. Sastry S. S. Sastry S. S. (1994). A mathematical introduction to robotic manipulation. CRC press.

[B34] Mussa-Ivaldi F. A. (1999). Modular features of motor control and learning. Curr. Opin. Neurobiol. 9, 713–717. 10.1016/s0959-4388(99)00029-x 10607638

[B35] Naceri A. Santello M. Moscatelli A. Ernst M. O. (2016). “Digit position and force synergies during unconstrained grasping,” in Human and Robot Hands, eds Bianchi M. Moscatelli A. (Switzerland: Springer International Publishing), 29–40. 10.1007/978-3-319-26706-7

[B36] Nowak D. A. Glasauer S. Hermsdörfer J. (2004). How predictive is grip force control in the complete absence of somatosensory feedback? Brain 127, 182–192. 10.1093/brain/awh016 14570822

[B37] Overduin S. A. d’Avella A. Carmena J. M. Bizzi E. (2014). Muscle synergies evoked by microstimulation are preferentially encoded during behavior. Front. Comput. Neurosci. 8:20. 10.3389/fncom.2014.00020 24634652 PMC3942675

[B38] Piazza C. Della Santina C. Catalano M. Grioli G. Garabini M. Bicchi A. (2016). “Softhand pro-d: matching dynamic content of natural user commands with hand embodiment for enhanced prosthesis control,” in Robotics and Automation (ICRA), 2016 IEEE International Conference on (Stockholm: IEEE), 3516–3523.

[B39] Puhlmann S. Heinemann F. Brock O. Maertens M. (2016). “A compact representation of human single-object grasping,” in Intelligent Robots and Systems (IROS), 2016 IEEE/RSJ International Conference on (Daejeon: IEEE), 1954–1959.

[B40] Saltiel P. Wyler-Duda K. D’Avella A. Tresch M. C. Bizzi E. (2001). Muscle synergies encoded within the spinal cord: evidence from focal intraspinal NMDA iontophoresis in the frog. J. Neurophysiol. 85, 605–619. 11160497 10.1152/jn.2001.85.2.605

[B41] Santello M. Baud-Bovy G. Jörntell H. (2013). Neural bases of hand synergies. Front. Comput. Neurosci. 7:23. 10.3389/fncom.2013.00023 23579545 PMC3619124

[B42] Santello M. Bianchi M. Gabiccini M. Ricciardi E. Salvietti G. Prattichizzo D. (2016). Hand synergies: integration of robotics and neuroscience for understanding the control of biological and artificial hands. Phys. Life Rev. 17, 1–23. 10.1016/j.plrev.2016.02.001 26923030 PMC5839666

[B43] Santello M. Flanders M. Soechting J. F. (1998). Postural hand synergies for tool use. J. Neurosci. 18, 10105–10115. 9822764 10.1523/JNEUROSCI.18-23-10105.1998 PMC6793309

[B44] Santello M. Flanders M. Soechting J. F. (2002). Patterns of hand motion during grasping and the influence of sensory guidance. J. Neurosci. 22, 1426–1435. 11850469 10.1523/JNEUROSCI.22-04-01426.2002 PMC6757566

[B45] Schafer R. W. (2011). What is a savitzky-golay filter? IEEE Sig. Process. Mag. 28, 111–117. 10.1109/msp.2011.941097

[B46] Schäling B. (2011). The boost C++ libraries. Boris Schäling.

[B47] Scholz J. P. Schöner G. (1999). The uncontrolled manifold concept: identifying control variables for a functional task. Exp. Brain Res. 126, 289–306. 10.1007/s002210050738 10382616

[B48] Serio A. Bianchi M. Gabiccini M. Bicchi A. (2014). “[d94] the tactile toolbox,” in Haptics Symposium (HAPTICS), 2014 IEEE (Houston: IEEE), 1.

[B49] Sircoulomb V. Hoblos G. Chafouk H. Ragot J. (2008). “State estimation under nonlinear state inequality constraints. a tracking application,” in Control and Automation, 2008 16th Mediterranean Conference on (Ajaccio: IEEE), 1669–1674.

[B50] Stratmann P. Lakatos D. Albu-Schäffer A. (2016). Neuromodulation and synaptic plasticity for the control of fast periodic movement: energy efficiency in coupled compliant joints via PCA. Front. Neurorobot. 10 10.3389/fnbot.2016.00002 PMC4782012 27014051

[B51] Thakur P. H. Bastian A. J. Hsiao S. S. (2008). Multidigit movement synergies of the human hand in an unconstrained haptic exploration task. J. Neurosci. 28, 1271–1281. 10.1523/jneurosci.4512-07.2008 18256247 PMC6671569

[B52] Tresch M. C. Saltiel P. Bizzi E. (1999). The construction of movement by the spinal cord. Nat. Neurosci. 2, 162–167. 10.1038/5721 10195201

[B53] Villani L. Ficuciello F. Lippiello V. Palli G. Ruggiero F. Siciliano B. (2012). “Grasping and control of multi-fingered hands,” in Advanced Bimanual Manipulation, 219–266. Springer.

[B54] Westling G. Johansson R. S. (1987). Responses in glabrous skin mechanoreceptors during precision grip in humans. Exp. Brain Res. 66, 128–140. 10.1007/bf00236209 3582527

[B55] Xu K. Liu H. Du Y. Zhu X. (2014). Design of an underactuated anthropomorphic hand with mechanically implemented postural synergies. Adv. Robot. 28, 1459–1474. 10.1080/01691864.2014.958534

